# Blood volume and albumin transudation in critically ill COVID-19 patients

**DOI:** 10.1186/s13054-021-03699-y

**Published:** 2021-07-31

**Authors:** Jan Bakker, James M. Horowitz, Jackie Hagedorn, Sam Kozloff, David Kaufman, Ricardo Castro

**Affiliations:** 1 Division of Pulmonary, Critical Care, and Sleep Medicine, New York University School of Medicine, New York, NY USA; 2 Division of Pulmonary, Allergy, and Critical Care Medicine, Columbia University College of Physicians and Surgeons, New York, NY USA; 3 Department of Intensive Care Adults, Erasmus MC University Medical Center, Rotterdam, Netherlands; 4 Department of Intensive Care, Pontificia Universidad Católica de Chile, Santiago, Chile; 5 Division of Cardiology, New York University School of Medicine, New York, NY USA

*To the Editor*:

The SARS-CoV-2 infection (COVID-19) in critically ill patients presents as a viral pneumonia and inflammation affecting the endothelium [1] with unclear consequences for fluid leakage to the extravascular space. Nevertheless, the adapted Surviving Sepsis Guidelines advocate a conservative fluid strategy [2]. By using a radiolabeled albumin tracer, the total blood volume (TBV), red blood cell volume (RBCV), plasma volume (PV), and the albumin transudation rate (ATR) can be measured [3]. In six mechanically ventilated patients (admitted March/April 2020), the TBV was measured [4] as advanced hemodynamic monitoring was not used, and the volume status was unclear. The volumes measured were corrected for the ideal body weight of a corresponding healthy individual, and deviations were calculated. The results of only the TBV, RBCV, and PV were communicated with the treatment team.

We retrospectively analyzed these data together with the ATR. Albumin transudation rate is presented as a numeric value with 0.0025 (0.25%/min exiting the circulation) serving as the normal reference threshold. We report absolute variation (values at admission minus value at day of measurement for each case), and day-indexed values, calculated by dividing the absolute variation by the number of days in the ICU. We performed univariate regression between albumin transudation and variables of interest. In the multivariate regression, we tested variables that showed statistical significance in the univariate analysis and other that did not reach the significance threshold but had clinical relevance. Data are expressed as mean ± 1 standard deviation unless otherwise indicated. A *p* value of equal or less than 0.05 was considered significant. None of the patients was diagnosed with a secondary infection the days before the measurement. Age of the patients was 66 ± 11 year with a mean weight of 86.3 ± 15.7 kg. Only one patient did not have any comorbidity on admission where the most frequent comorbidity was diabetes (four patients, two in combination with hypertension). Four patients died, all of whom developed complete renal failure. At the time of measurement, all patients had stable hemodynamics only one patient received vasopressor support (norepinephrine 0.42 mcg/kg/min). Results are shown in Table 1. Three of the four clinically hypervolemic patients (assessed by fluid balance and extend of peripheral edema), TBV showed a decreased value from ideal body weight. The median ATR was 0.46%/min (range 0.12–0.82). There was a strong linear relationship between the day of admission and ATR (R^2 ^= 0.99, *P* < 0.0001) and a curve linear relationship with the deviation of the TBV (R^2^ = 0.63, *p* < 0.03) (Fig. 1).

**Table 1:** Characteristics of individual patients at day of blood volume measurement

Parameter at time of measurement	Pat 1 #S	Pat 2 %#NS	Pat 3 NS	Pat 4 NS	Pat 5 #NS	Pat 6 S	Mean ± SD Median IQR
ICU Admission	March 2020	March 2020	April 2020	April 2020	April 2020	April 2020	
Day of ICU admission at measurement	14	20	11	13	7	2	
Age|male/female	70|M	69|M	49|M	62|M	66|F	81|M	66 ± 11 68 (59–73)
Height (cm)	1.75	1.73	1.65	1.73	1.58	1.52	166 ± 0.09 | 1.69 (1.57–1.74)
Weight (kg)	76.2	78.0	77.8	67.8	93.9	61.8	75.9 ± 10.9 | 77.0 (66.3–82.0)
Ideal body weight (kg)	70.0	69.0	61.0	69.0	51	50.0	61.7 ± 9.2 | 65.0 (50.8–69.3)
Heart rate (b/min)	95	104	97	69	112	72	92 ± 17 96 (71–106)
Systolic arterial pressure (mmHg)	137	72	97	94	146	96	107 ± 28 97 (89–138)
Diastolic arterial pressure (mmHg)	56	38	66	48	52	42	50 ± 10 50 (41–59)
Mean arterial pressure (mm Hg)	79	50	76	66	75	58	67 ± 11 71 (56–77)
Lactate (mmol/L)	1.5	1.6	1.3	2.5	1.8	1.4	1.7 ± 0.4 1.6 (1.4–2.0)
C reactive protein at measurement (mg/L)	3	239	409	282	263	50	208 ± 153 251 (38–313)
Inspired oxygen fraction	0.4	0.8	0.7	1	0.8	0.4	0.68 ± 0.24 0.75 (0.4–0.9)
Pulse oximetry (%)	97	95	96	95	91	96	95 ± 2 96 (94–97)
Net fluid balance day before measurement (L)	− 0.9	1.2	1.3	− 0.3	− 0.9	3.0	0.6 ± 1.5 0.45 (− 0.9 to 1.7)
Fluids IN day before measurement (L)	3.6	2.1	2.6	1.7	3.1	3.2	2.7 ± 0.7 2.9 (2.0–3.3)
Urine output day before measurement (L)	4.5	0.7	0.8	2.7	1.8	0.7	1.9 ± 1.5 1.3 (0.7–3.2)
Fluids in since admission (L)	36.8	41	17.2	19.8	15.4	3.8	22.3 ± 14.0 18.5 (12.5–37.9)
Net fluid balance since admission (L)	8.2	10	5.9	3.0	0.3	4.0	5.2 ± 3.5 5.0 (2.3–8.7)
Clinical assessment of volume status	Hyper	Hyper	Eu	Hyper	Hyper	Hypo	
Total blood volume (mL)	4200	4290	5360	3990	4870	4552	4544 ± 502 4421 (4223–4791)
Red cell volume (mL)	1215	935	1391	841	1220	1370	1162 ± 227 1218 (1005–1333)
Plasma volume (mL)	2985	3355	3969	3149	3650	3182	3382 ± 366 3269 (3157–3576)
Total blood volume dev (%)	− 16.6	− 17.3	− 15.2	− 18.9	+ 3.3	+ 17.2	− 7.9 ± 14.8 − 15.9 (− 17.7 to 6.8)
Red cell volume dev (%)	− 41%	− 56%	− 26%	− 58%	− 28%	− 13%	− 34 (− 56 to − 23)
Plasma volume dev (%)	− 0%	9%	44%	8%	21%	38%	15% (6–39)
Albumin transudation rate (%/min)	0.58	0.82	0.43	0.49	0.24	0.12	0.45 ± 0.25 0.46 (0.21–0.64)

Mean ± SD and median (IQR 25, 75)

% = being treated with norepinephrine, # = being treated with diuretic, S = survivor, NS = non-survivor

Hyper: hypervolemia, Eu: euvolemia, Hypo: hypovolemia. Total blood volume deviation: absolute and relative deviation of the expected total blood volume of a healthy individual

**Fig. 1:**
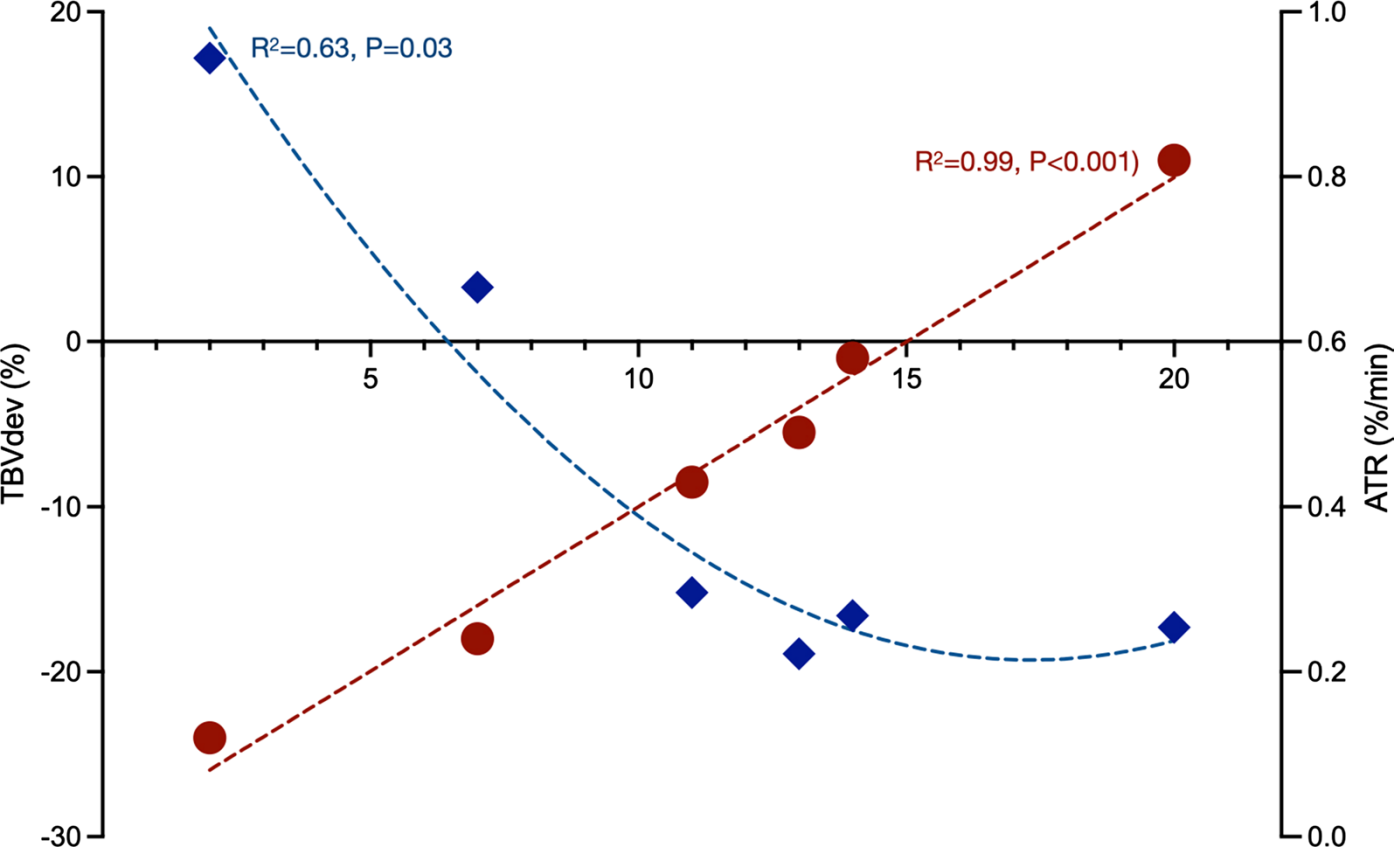
Relationship between the albumin transudation rate (ATR) and the deviation of the total blood volume from ideal (TBV deviation)

In an exploratory multivariate regression model, we found TBV deviation (*p* = 0.022) and net fluid balance since admission (*p* = 0.018) and CRP at day of measurement (*p* = 0.043) to explain 99% of the variation in ATR.

Although endothelial damage is believed to be an important part of COVID-19 and related to the severity of illness [5] and aggregates of red and white blood cells in the microcirculation have been reported [6], extensive capillary leakage has not been reported before. As a nosocomial bacterial infection had not been confirmed in any of the patients, the severity of COVID-19 and the systemic inflammatory response could be a more likely explanation. This vascular leakage could result in tissue edema and ultimately organ dysfunction as seen in these patients. Although this may suggest a role for the use of specific fluids (such as colloids) in this disease, our current data do not allow such recommendation. Given the only one-time measurement results should be interpreted with caution. This study could be seen as a unique exploratory study in COVID-19 patients. We have therefore initiated a multicenter prospective study to improve our understanding of blood volume and vascular leakage in critically ill COVID-19 patients (NCT04517695).

## Data Availability

Supporting data are available upon request to the corresponding author 3 months after publication.
